# Smoking and risk of incident end-stage kidney disease in general population: A Nationwide Population-based Cohort Study from Korea

**DOI:** 10.1038/s41598-019-56113-7

**Published:** 2019-12-20

**Authors:** Hong Sang Choi, Kyung-Do Han, Tae Ryom Oh, Chang Seong Kim, Eun Hui Bae, Seong Kwon Ma, Soo Wan Kim

**Affiliations:** 1 Department of Internal Medicine, Chonnam National University Medical School, Gwangju, 61469 Korea; 2 Department of Medical Statistics, College of Medicine, The Catholic University of Korea, Seoul, 06591 Korea

**Keywords:** End-stage renal disease, Renal replacement therapy

## Abstract

We analyzed data from the Korean National Health Insurance Service (NHIS) to investigate whether smoking increases the risk of end-stage kidney disease (ESKD). This retrospective nationwide population-based cohort study included the data of 23,232,091 participants who underwent at least one health examination between 2009 and 2012. Smoking status was recorded at baseline. The incidence of ESKD was identified via ICD-10 codes and special medical aid codes from the Korean National Health Insurance Service database till December 2016. A Cox proportional-hazards model with multivariable adjustment was used to evaluate the association between smoking and ESKD incidence. Overall, 24.6% of participants were current smokers; 13.5% and 61.9%, were ex- and non-smokers, respectively. Overall, 45,143 cases of ESKD developed during the follow-up period. Current smokers (hazard ratio [HR], 1.39; 95% confidence interval [CI], 1.35–1.43) and ex-smokers (HR, 1.09; 95% CI, 1.06–1.12) demonstrated a significant increase in the adjusted risk of ESKD compared to non-smokers. The risk of ESKD was directly proportional to the smoking duration, number of cigarettes smoked daily, and pack-years. In conclusion, smoking is associated with a greater risk of ESKD in the general Korean population; the risk increases with an increase in the smoking duration, number of cigarettes smoked daily, and pack-years.

## Introduction

Smoking is known to be one of the leading causes of preventable deaths worldwide^1,2^, with more than seven million deaths caused by tobacco use every year^3^. Smoking increases the risk of various chronic diseases including cancer, cardiovascular disease, and respiratory disease^4–7^; it may also increase the risk of developing chronic kidney disease (CKD)^7,8^. Although efforts have been made to recognize the hazards of smoking and to reduce the number of smokers, their numbers continue to increase, particularly in low - income countries^3^.

CKD is a growing global public health problem, with an estimated global prevalence of 8–16%^9^. The prevalence and incidence of end-stage kidney disease (ESKD), which is caused by progression of CKD, differs among different countries and regions. In Korea, the numbers of patients treated with renal replacement therapy are approximately 98,000, and the numbers of new ESKD patients are rapidly increasing every year^10^. Therefore, early diagnosis and treatment, and the prevention of CKD by managing risk factors in particular, is becoming an important public health concern.

Previous studies have reported on the impact of smoking status on ESKD incidence in the general population^11–17^. However, large cohorts have never been analyzed on a national scale. To better understand the association between smoking and adverse renal outcomes in the broader general population, we analyzed nationally representative data from the Korean National Health Insurance System (NHIS).

## Results

### Baseline characteristics of the study population

The baseline clinical characteristics of the population are shown in Table 1. At baseline, 14,380,975 (61.9%), 3,145,747 (13.5%), and 5,705,969 (24.6%) participants were never, ex-, and current smokers, respectively. Compared with never smokers, the current smokers were younger and were more likely to be male. In particular, current and ex-smokers were mostly males, while approximately 25% of never smokers were male. Current smokers were also more likely to have a higher BMI and waist circumference, be current drinkers, and have diabetes than never smokers. Current smokers were less likely than never smokers to have hypertension, dyslipidemia, and prior history of stroke, heart diseases and CKD. Ex-smokers were more likely to have hypertension, diabetes, dyslipidemia, and a history of stroke and heart disease than current smokers.

**Table 1:** Baseline clinical characteristics of study population according to the smoking status.

	Never smokers	Ex-smokers	Current smokers
	(n = 14,380,975; 61.9%)	(n = 3,145,147; 13.5%)	(n = 5,705,969; 24.6%)
Male sex (%)	3,625,811 (25.2)	2,913,030 (92.6)	5,240,583 (91.8)
Age (years)	48.9 ± 14.8	50.1 ± 13.4	43.4 ± 12.9
**Age categories**			
20–39	3,689,380 (25.7)	700,064 (22.3)	2,353,989 (41.3)
40–64	8,322,049 (57.9)	1,962,144 (62.4)	2,957,639 (51.8)
≥65	2,369,546 (16.5)	482,939 (15.4)	394,341 (6.9)
**Alcohol consumption**			
Non-drinker	10,046,912 (69.9)	1,005,767 (32.0)	1,398,246 (24.5)
Up to 30 g/day	4,062,366 (28.3)	1,773,883 (56.4)	3,408,447 (59.7)
More than 30 g/day	271,697 (1.9)	365,497 (11.6)	899,276 (15.8)
Regularly exercise	6,578,023 (45.7)	1,948,877 (62.0)	2,992,952 (52.5)
Body mass index (kg/m^2^)	23.5 ± 3.3	24.4 ± 3.0	23.9 ± 3.3
**BMI categories**			
<18.5 kg/m^2^	680,982 (4.7)	61,798 (2.0)	198,756 (3.5)
18.5–23 kg/m^2^	6,143,663 (42.7)	959,450 (30.5)	2,131,459 (37.4)
23–25 kg/m^2^	3,340,464 (23.2)	877,229 (27.9)	1,401,449 (24.6)
25–30 kg/m^2^	3,705,300 (25.8)	1,134,436 (36.1)	1,735,753 (30.4)
≥30 kg/m^2^	510,566 (3.6)	112,234 (3.6)	238,552 (4.2)
Waist circumference (cm)	78.1 ± 9.3	84.1 ± 8.1	82.6 ± 8.5
Systolic BP (mmHg)	121.2 ± 15.6	125.2 ± 14.6	123.4 ± 14.2
Diastolic BP (mmHg)	75.1 ± 10.1	78.1 ± 9.9	77.3 ± 9.9
Hypertension	3,768,218 (26.2)	1,052,872 (33.5)	1,295,427 (22.7)
Diabetes mellitus	1,226,829 (8.5)	390,300 (12.4)	538,169 (9.4)
Dyslipidemia	2,842,502 (19.8)	683,577 (21.7)	949,154 (16.6)
History of stroke	131,823 (1.4)	57,751 (2.7)	42,640 (1.2)
History of heart disease	295,745 (3.2)	108,666 (5.1)	76,822 (2.1)
Chronic kidney disease	923,335 (6.4)	174,041 (5.5)	179,267 (3.1)
Hemoglobin (g/dL)	13.3 ± 1.5	14.7 ± 1.3	15.0 ± 1.3
Fasting glucose (mg/dL)	96.4 ± 21.9	101.0 ± 25.1	98.7 ± 25.8
Total cholesterol (mg/dL)	194.7 ± 37.0	195.5 ± 36.5	194.3 ± 36.8
Proteinuria	350,718 (2.4)	91,457 (2.9)	141,676 (2.5)

Data are expressed as the mean ± SD, or n (%).

BP, blood pressure; SD, standard deviation.

### Risk of ESKD by smoking status

During the follow-up period (mean 6.35 ± 1.17, median 6.59 years), a total of 45,143 incident cases of ESKD occurred. The incidence rate (IR) of ESKD was highest in ex-smokers (0.45278 event per 1000 person-years), and similar in non-smokers and current smokers (0.28386 and 0.28093 per 1000 person-years, respectively; Table 2). Compared with non-smokers, the ESKD event risk was 12% higher among ex-smokers (HR, 1.12; 95% CI, 1.09–1.15) and 10% higher among current smokers (HR, 1.10; 95% CI, 1.07–1.13) when adjusted for age and sex, respectively. After full adjustment, the ESKD event risk was 9% higher among ex-smokers (HR, 1.09; 95% CI, 1.06–1.12) and 39% higher among current smokers (HR, 1.39; 95% CI, 1.35–1.43). In male participants, ex-smokers and current smokers had a higher risk of ESKD incidence when compared with never smokers (HR, 1.07; 95% CI 1.04–1.10 and HR, 1.34; 95% CI 1.30–1.38, respectively). Notably, in females, both ex- and current smokers had a greater increase in the risk of ESKD (HR, 1.46; 95% CI, 1.31–1.62 and HR, 1.58; 95% CI, 1.46–1.71, respectively). On competing risk analysis, which also considered the risk of death, a significant association persisted between smoking and an increased risk of developing ESKD in all groups except male ex-smokers (Table 2).

**Table 2:** Multivariate Cox regression analyses for ESKD development.

		Follow-up duration (person-years)	Incident ESKD	Incidence rate (per 1000 person-years)	Crude HR (95% CI)	Adjusted HR (95% CI)		
						Model 1^a^	Model 2^b^	Model 3^c^
Total	Never-smoker	91,301,111.65	25,917	0.28386	1 (ref.)	1 (ref.)	1 (ref.)	1 (ref.)
	Ex-smoker	20,084,800.68	9,094	0.45278	1.59 (1.55, 1.63)	1.12 (1.09, 1.15)	1.09 (1.06. 1.12)	1.07 (1.00, 1.13)
	Current smoker	36,065,551.85	10,132	0.28093	0.99 (0.97, 1.01)	1.10 (1.07, 1.13)	1.39 (1.35, 1.43)	1.37 (1.29, 1.45)
Male	Never-smoker	231,22,752.33	9,690	0.41907	1 (ref.)	1 (ref.)	1 (ref.)	1 (ref.)
	Ex-smoker	18,661,343.11	8,732	0.46792	1.12 (1.08, 1.15)	1.09 (1.06, 1.12)	1.07 (1.04. 1.10)	1.05 (0.98, 1.11)
	Current smoker	33,245,852.3	9,434	0.28376	0.68 (0.66, 0.70)	1.09 (1.06, 1.12)	1.34 (1.30, 1.38)	1.34 (1.26, 1.43)
Female	Never-smoker	68,178,359.31	16,227	0.23801	1 (ref.)	1 (ref.)	1 (ref.)	1 (ref.)
	Ex-smoker	1,423,457.58	362	0.25431	1.08 (0.97, 1.20)	1.54 (1.39, 1.71)	1.46 (1.31, 1.62)	1.47 (1.21, 1.80)
	Current smoker	2,819,699.55	698	0.24754	1.06 (0.98, 0.14)	1.37 (1.27, 1.48)	1.58 (1.46, 1.71)	1.38 (1.16, 1.64)

^a^Adjusted for age, sex.

^b^Adjusted for age, sex, body mass index, alcohol consumption, regular exercise, income, diabetes mellitus, hypertension, chronic kidney disease, dyslipidemia, hemoglobin level and proteinuria.

^c^Model 2+ competing risk model.

Abbreviation: HR, hazard ratio; CI, confidence interval; ref, reference.

### Risk of ESKD by smoking duration, daily amounts and pack-years

**Figure 1:**
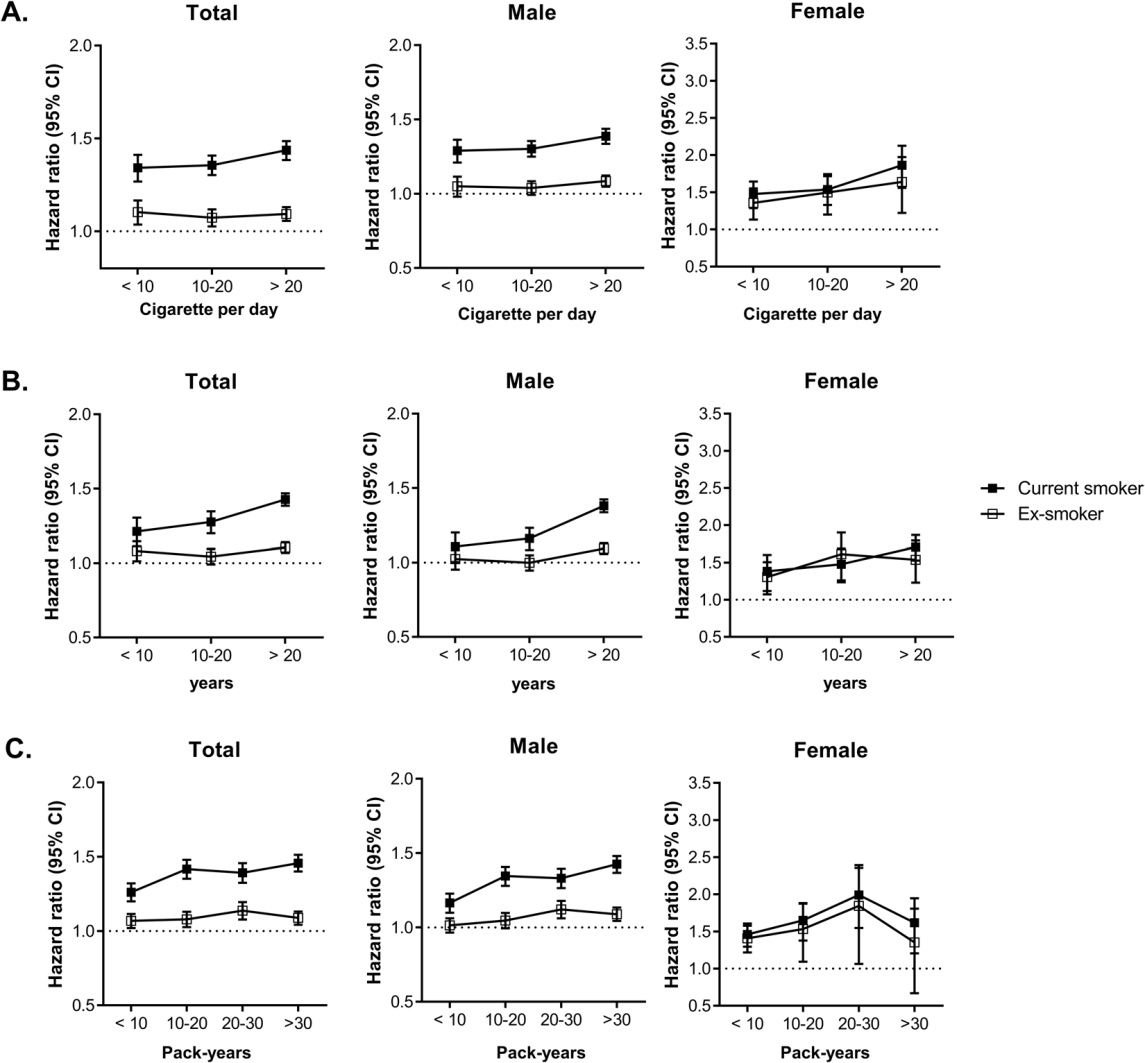
Relevance of smoking status to risk of incident ESKD. (**A**) Amount of cigarette per day. (**B**) Duration of smoking (years) and (**C**) pack-years. Hazard ratio of incident ESKD according to smoking status. Error bars represent 95% confidence intervals for lower and upper limits. Adjusted for age, sex, body mass index, alcohol consumption, regular exercise, income, diabetes mellitus, hypertension, chronic kidney disease, dyslipidemia, hemoglobin level and proteinuria.

We analyzed the impact of various indicators of smoking, amount of daily smoking, duration of smoking, and pack-years on the risk of developing ESKD (Fig. 1). Overall, the risk of ESKD significantly increased with an increase in the number of cigarettes smoked per day, smoking duration, and pack-years; this trend was more prominent among the current smokers. In the total population and among males, the risk of ESKD was higher in all subgroups of current smokers when stratified by the number of cigarettes smoked per day, duration of smoking, and pack-years than in the corresponding ex-smoker subgroups. Among female ex-smokers, the risk of ESKD was not significantly different from that of non-smokers in the group with the highest pack-years.

### Risk of ESKD by disease subgroups

Subgroup analysis was performed by factors known to be associated with increased risk of ESKD, namely, age, sex, alcohol consumption, regular exercise, obesity, diabetes, hypertension, CKD and dyslipidemia (Table 3). Current smokers showed a significantly higher risk for developing ESKD in all subgroups compared with the never smokers. Higher adjusted HRs of incident ESKD were observed in the subgroup that was older (>65 years), not obese, and without diabetes mellitus, hypertension, CKD, dyslipidemia, or proteinuria (p for interaction <0.001). The associations between smoking status and ESKD were consistent irrespective of drinking status or exercise (p for interaction 0.103 or 0.315, respectively). Ex-smokers also showed a significant increase in the risk of ESKD compared to never smokers in almost all subgroups.

**Table 3:** Hazard ratio and 95% confidence intervals of incident ESKD in subgroups.

	Subgroup	HR^a^ (95% CI)			*p* for interaction
		Never smoker	Ex-smoker	Current smoker	
Age, years	20–39	1 (ref.)	1.17 (1.02, 1.33)	1.39 (1.24, 1.54)	<0.001
	40–64	1 (ref.)	1.02 (0.98, 1.06)	1.33 (1.28, 1.39)	
	65-	1 (ref.)	1.12 (1.08, 1.17)	1.42 (1.36, 1.49)	
Alcohol consumption	Non-drinker	1 (ref.)	1.11 (1.07, 1.15)	1.36 (1.31, 1.41)	0.103
	≤30 g/day	1 (ref.)	1.06 (1.01, 1.12)	1.45 (1.38, 1.53)	
	>30 g/day	1 (ref.)	1.01 (0.88, 1.15)	1.39 (1.23, 1.57)	
Regular exercise	no	1 (ref.)	1.08 (1.04, 1.12)	1.38 (1.33, 1.44)	0.315
	yes	1 (ref.)	1.10 (1.05, 1.14)	1.40 (1.34, 1.46)	
Obesity	Non-obese	1 (ref.)	1.09 (1.05, 1.13)	1.40 (1.35, 1.45)	<0.001
	obese	1 (ref.)	1.08 (1.03, 1.13)	1.34 (1.27, 1.40)	
Diabetes mellitus	No	1 (ref.)	1.15 (1.10, 1.19)	1.41 (1.36, 1.47)	<0.001
	Yes	1 (ref.)	1.02 (0.99, 1.07)	1.29 (1.24, 1.34)	
Hypertension	No	1 (ref.)	1.13 (1.06, 1.21)	1.35 (1.27, 1.43)	<0.001
	Yes	1 (ref.)	1.06 (1.03, 1.10)	1.34 (1.30, 1.38)	
Chronic kidney disease	No	1 (ref.)	1.10 (1.05, 1.15)	1.47 (1.41, 1.54)	<0.001
	Yes	1 (ref.)	1.06 (1.03, 1.10)	1.31 (1.26, 1.36)	
Dyslipidemia	No	1 (ref.)	1.12 (1.07, 1.16)	1.42 (1.37, 1.47)	<0.001
	Yes	1 (ref.)	1.06 (1.01, 1.10)	1.33 (1.27, 1.39)	
Proteinuria	No	1 (ref.)	1.15 (1.10, 1.19)	1.49 (1.43, 1.55)	<0.001
	Yes	1 (ref.)	1.02 (0.98, 1.06)	1.28 (1.22, 1.33)	

^a^Adjusted for age, sex, body mass index, alcohol consumption, regular exercise, income, diabetes mellitus, hypertension, chronic kidney disease, dyslipidemia, hemoglobin level and proteinuria.

Abbreviations: BMI. Body mass index; HR, hazard ratio; CI, confidence interval.

## Discussion

In this retrospective cohort study, we found that smoking was closely associated with an increased risk of incident ESKD cases in the adult general population. Current and ex-smokers showed a significantly increased risk of developing ESKD compared to never smokers. These associations interacted with age, sex, BMI, and presence of diabetes, hypertension, CKD, and dyslipidemia. The impact of smoking on the development of ESKD was elevated with an increased amount of smoking, duration of smoking, and pack-years.

The baseline clinical characteristics demonstrated the proportions of male current and ex-smokers to be extremely high. Compared with other previously published studies, the proportion of males among smokers was very high^13,18^. This was thought to be partly influenced by Korea’s unique culture, which strongly discourages smoking among women. It is also possible that women did not openly declare their smoking status in the health screening questionnaire in view of the potential consequences of being identified as a smoker; the smoking prevalence of females was therefore likely to be underestimated or underreported. In addition, the prevalence of various diseases except diabetes, was higher in current smokers than in non-smokers. Conversely, it was thought that relatively healthy participants without underlying disease are likely to be current smokers. However, ex-smokers had a significantly higher prevalence of various diseases than current smokers. This was thought to be caused by smoking cessation for health after diagnosis of the disease. The different tendency in CKD could be explained by the fact that the patient does not recognize early CKD in many cases.

Table 2 demonstrates that the risk of ESKD in current and ex-smokers was higher compared to that of non-smokers. Compared to never smokers, the risk of ESKD was 9% and 39% higher in ex- and current smokers, respectively. These results were consistent with those of previous studies in the general population^11–17^. A study on more than 65,000 participants in a Norwegian health survey cohort revealed that ex- and current smokers had an approximately 3.3- and 4.0-fold increased risk of kidney failure, respectively, compared to never smokers^14^. A study on 63,000 Singapore Chinese health study participants showed that ex- and current smokers had a 1.42- and 1.28-fold risk of kidney failure, respectively, compared to never smokers^16^. In our study, ex- and current smokers were 1.09 and 1.39 times more likely to develop ESKD compared to never smokers, respectively. Smoking was also found to significantly increase the risk of developing ESKD on competing risk analysis (HR 1.07 and 1.37 in ex-smokers and current smokers, respectively). In particular, the increased risk of ESKD in current smokers was consistent with the findings of most studies^11–14,16^. However, the findings among ex-smokers slightly differed from those of previous studies^12–14,16^. A recently published meta-analysis did not demonstrate a significant increase in the risk of ESKD in ex-smokers (relative risk 1.44, 95% CI, 0.99–2.09)^19^. Based on a considerably larger number and wider range of participants than in previous studies, our study showed that in addition to current smokers, ex-smokers also had an increased risk of ESKD in the general population.

However, the incidence ratio of ESKD was higher in ex-smokers than in current smokers; this was most likely to be due to the higher prevalence of underlying diseases such as diabetes and hypertension, which are considered to be risk factors for ESKD in ex-smokers. After adjustment for these variables, the risk of ESKD was highest in current smokers. On analyzing separately according to sex in both, current and ex-smokers, the risk of ESKD increase was greater in women than in men. Previous studies had reported a higher risk of ESKD incidence in men compared to women; the findings of our study differed^13,14,16^. The reason for the discrepancy between our and other studies remains unclear. However, this may be attributed to the fact that smoking was probably underreported in women. It was also possible that this is a statistical problem caused by a significantly lower number of smokers in women than non-smokers.

We also analyzed the risk of developing ESKD according to the amount of daily smoking, smoking duration, and pack-years. For each quantitative indicator of smoking, the risk of ESKD increased in a dose-response relationship, and was more prominent in males. Two previous studies had analyzed the risk of kidney failure based on the indicators of smoking, and reported results that were similar to ours^14,16^. We further analyzed the risk of ESKD and considered the current smoking status based on all indicators of smoking. Remarkably, in female ex-smokers, the group with the longest smoking period and the highest pack-years did not show an increased risk of ESKD compared to non-smokers. Potentially, this group of females could include relatively healthy elderly women, who have been smoking for a long time.

Subgroup analysis showed an increased risk of ESKD in almost all subgroups. However, in the association between smoking and the risk of ESKD incidence, there were considerable interactions with factors such as age, obesity, hypertension, diabetes, chronic kidney disease, hyperlipidemia, and proteinuria. Smoking was associated with an increased risk of developing ESKD irrespective of the drinking status or physical exercise. Previous studies have shown that cigarette smoking does not significantly increase the risk of ESKD in CKD participants^18^. In our study, however, the ESKD risk in those with CKD was relatively lower than that of non-CKD participants (p for interaction <0.001); however, compared to never smokers, there was a statistically significant increase in risk in both, ex- and current smokers.

Smoking is known to increase urine albumin excretion and blood pressure, and affect intrarenal hemodynamics^20–22^. It is known that pathophysiologically, smoking induces inflammation, oxidative stress, and endothelial dysfunction^23–25^. Studies show that smoking increases the activity of superoxide dismutase, significantly increasing kidney fibrosis in rats exposed to smoke. In a study, the kidneys of rats exposed to smoke showed an increase in transforming growth factor beta, which is known to be a critical mediator of renal fibrosis^25^. Additionally, nicotine induces podocyte apoptosis *in vitro* through reactive oxygen species generation and associated downstream mitogen-activated protein kinase signaling^26^. Smoking can also induce insulin resistance^27,28^ and generate advanced glycation end products, which ultimately result in renal damage^29^. In a cohort study on 2,490 diabetic participants, smoking showed a dose- and time-dependent relationship with glycemic control and insulin resistance^28^. In view of these findings, smoking is believed to accelerate the progression of CKD; we have demonstrated this using large-scale population data. In a recent study on the Korean population, secondhand smoke had also been reported to increase the risk of CKD progression^30^. On cross-sectional analysis, the risk of having CKD was significantly higher, and in those who experienced secondhand smoke, the longitudinal study showed an increase in the risk of developing CKD by more than 50%.

Our study has certain limitations. First, the retrospective and observational design of the study is a limitation. Second, the smoking status used in this study was self-reported at baseline, and did not account for changes in smoking habits over the course of the follow-up period. In particular, as mentioned previously, smoking in women was likely to be underestimated or underreported. Third, our study did not evaluate the drugs used. There may have been effects from renoprotective or nephrotoxic medications. Nevertheless, our study has several strengths compared to others. The key strengths of this study include the enrollment of the largest known study population of 23,232,091 participants, a wide age range, and a relatively long-term follow-up period. In addition, dose-response relationships between smoking and ESKD were explained using detailed smoking indices. A dose-response relationship is usually considered to be evidence for supporting causality.

In conclusion, smoking increases the risk of ESKD in the general population. The importance of smoking has been undervalued compared to the major risk factors of renal failure such as diabetes and hypertension. However, as shown in our study, smoking is also an important risk factor for the development of ESKD; careful management is therefore necessary. National smoking management and policies are essential for reducing the incidence of smoking-related diseases including ESKD, and to reduce the socio-economic and medical costs associated with smoking.

## Methods

### Data source and study population

Information regarding the Korean NHIS have been published previously^31,32^. Among the participants who received at least one health examination between 2009 and 2012, 23,452,862 were included after excluding duplicated data and participants under 20 years of age. The information regarding smoking status was reported in health examinations^33^. Smoking status was classified based on the health examination questionnaire records as follows: current smokers, defined as those who had smoked more than 5 packs (a total of 100 cigarettes) throughout their lifetime and continued to smoke, ex-smokers, defined as those who had smoked more than 5 packs (a total of 100 cigarettes) throughout their lifetime but had quit smoking, and never smokers, defined as those who had smoked 5 packs or fewer^34^. Both ex-smokers and current smokers recorded the total duration of smoking (years) and the average daily amount of cigarettes smoked (number of cigarettes per day) in the self-reporting questionnaire. The cumulative lifetime smoking exposure was reported as the pack-year by multiplying the average cigarette consumption per day (pack) by the smoking period (years).

**Figure 2:**
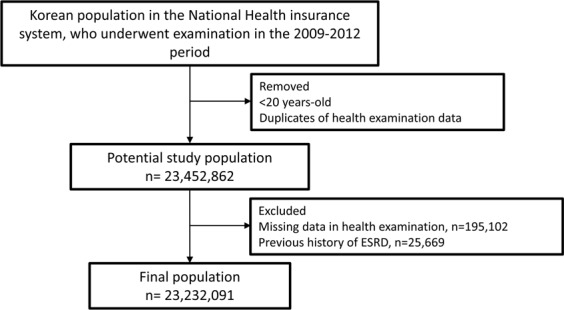
Flow diagram of study population selection.

After excluding people with missing data for health examinations (n = 195,102) and those with ESKD diagnosed before 2009 (n = 25,669), 23,232,091 participants without ESKD were followed-up from the January 2009 to the December 2016. Figure 2 shows a flowchart of the study design.

### Definitions of variables and outcomes

Data regarding smoking, status of alcohol consumption, and physical activities were obtained from health examination questionnaires. Standardized self-reported questionnaires were used for the following variables: alcohol consumption (none; mild, <30 g of alcohol/day; heavy, ≥30 g of alcohol/day), and smoking status (never, former, and current). Regular physical exercise was defined as high-intensity activity, ≥1 times/week or moderate-intensity activity, ≥1 times/week. The history of stroke and heart disease (particularly myocardial infarction and angina pectoris) was also obtained from questionnaires.

Body mass index (BMI) was calculated as the subject’s weight in kilograms divided by the square of the subject’s height in meters. Obesity was defined as BMI ≥25 kg/m^2^ ^35^. Abdominal obesity was defined as a waist circumference ≥90 cm for men and ≥85 cm for women. Blood samples for the measurement of serum creatinine, hemoglobin, total cholesterol and glucose were drawn after an overnight fast. Estimated glomerular filtration rate (eGFR) was calculated using the modification of diet in renal disease study (MDRD) formula and defined as CKD below eGFR 60 ml/min/1.73 m^2^. Proteinuria was defined as having urinary protein ≥1+ on dipstick testing in fasting morning urine. The above variables were extracted from health examination data provided to health insurance participants by NHIS biennially. Hospitals that performed these health examinations were certified by the NHIS and subjected to regular quality control evaluations.

The level of income was divided into quartiles, and the lowest quartile was defined as low income. The presence of diabetes mellitus was defined according to the following criteria: at least one claim per year under ICD-10 codes E11–14 and at least one claim per year for the prescription of antidiabetic medication or fasting serum glucose levels ≥126 mg/dl in the health examination database. The presence of hypertension was defined according to the presence of at least one claim per year under ICD-10 codes I10–I13 and I15 and at least one claim per year for the prescription of an antihypertensive agent or systolic/diastolic blood pressure ≥140/90 mmHg in the health examination database. The presence of dyslipidemia was defined according to the presence of at least one claim per year under ICD-10 code E78 and at least one claim per year for the prescription of a lipid-lowering agent or a total cholesterol level ≥240 mg/dL.

The study population was followed-up from the time of baseline measurement till the date of ESKD diagnosis, or until December 31, 2016, whichever came first. The primary endpoint was incident ESKD. The definition of ESKD have been published previously^31,32^.

### Statistical analyses

The baseline characteristics of participants have been presented as means ± standard deviation [SD] or n (%). The incidence rates of the primary outcomes were calculated by dividing the number of incident cases by the total length of the follow-up period. The incidence rates of the primary outcomes have been presented as per 1000 person-years. Hazard ratios (HRs) and 95% confidence interval (CI) values for the occurrence of ESKD among groups were analyzed using Cox proportional-hazards models. Multivariable-adjusted proportional-hazards models were applied as follows: model 1 was adjusted for age and sex, and model 2 was further adjusted for BMI, alcohol consumption, physical activity, hemoglobin level, lower income status, diabetes mellitus, hypertension, CKD, and proteinuria. Competing risk regression analysis was performed using the Fine and Grey model to account for the potential risk of death as a competing risk; we also performed subgroup analyses. The potential effect modification by age group, alcohol consumption status, regular exercise, presence or absence of obesity, diabetes mellitus, hypertension, dyslipidemia, CKD, and proteinuria were evaluated through stratified analysis and interaction testing using a likelihood ratio test. Statistical analyses were performed using SAS version 9.3 (SAS Institute, Cary, NC, USA), and a p value < 0.05 was considered to indicate statistical significance.

### Ethical approval

The requirement for ethical approval for this study was waived by the Institutional Review Board (IRB) of the Chonnam National University Hospital (#IRB No. CNUH-EXP-2018-232). The requirement for obtaining informed consent was also waived; hence, consent was not obtained as participants records and information were anonymized and de-identified prior to analysis.
